# ASimOV: A Framework for Simulation and Optimization of an Embedded AI Accelerator

**DOI:** 10.3390/mi12070838

**Published:** 2021-07-19

**Authors:** Dong Hyun Hwang, Chang Yeop Han, Hyun Woo Oh, Seung Eun Lee

**Affiliations:** Department of Electronic Engineering, Seoul National University of Science and Technology, 232 Gongneung-ro, Nowon-gu, Seoul 01811, Korea; hwangdonghyun@seoultech.ac.kr (D.H.H.); hanchangyeop@seoultech.ac.kr (C.Y.H.); ohhyunwoo@seoultech.ac.kr (H.W.O.)

**Keywords:** artificial intelligence, k-NN, embedded system

## Abstract

Artificial intelligence algorithms need an external computing device such as a graphics processing unit (GPU) due to computational complexity. For running artificial intelligence algorithms in an embedded device, many studies proposed light-weighted artificial intelligence algorithms and artificial intelligence accelerators. In this paper, we propose the ASimOV framework, which optimizes artificial intelligence algorithms and generates Verilog hardware description language (HDL) code for executing intelligence algorithms in field programmable gate array (FPGA). To verify ASimOV, we explore the performance space of k-NN algorithms and generate Verilog HDL code to demonstrate the k-NN accelerator in FPGA. Our contribution is to provide the artificial intelligence algorithm as an end-to-end pipeline and ensure that it is optimized to a specific dataset through simulation, and an artificial intelligence accelerator is generated in the end.

## 1. Introduction

The performance of the artificial intelligence algorithm is overwhelming compared with other algorithms. Due to the high performance of artificial intelligence algorithms, convolutional neural networks and recurrent neural networks are applied to other problem tasks, such as computer vision and time–series data processing [1,2,3,4]. Recently, the sophisticated results of artificial intelligence algorithms such as generative pretrained transformer (GPT), DeepFake, and Deep Voice have had a high social impact to the extent that problems of ethics arise [5,6,7]. The artificial intelligence algorithm has increased in computational complexity over time and computed a device such as a GPU to consume power, almost >100 W [8]. It is an obstacle that the artificial intelligence algorithm applies to the embedded system or mobile device.

To reduce the computational complexity of artificial intelligence algorithms, many studies propose lightweight artificial neural network algorithms. The pruning method re-moves unnecessary neurons from neural networks [9,10,11]. Knowledge distillations transfer refined knowledge of well-trained models to smaller, untrained models [12,13,14], and the bit quantization methods quantize 32-bit or 64-bit floating-point weights to 2 to 8-bit integer weights in a neural network [15,16,17]. To reduce power consumption, many studies propose the artificial neural network accelerator. FINN-R explores binary neural network architecture space and executes a binary neural network in Xilinx FPGA (Xilinx, Inc, San Jose, CA, USA) [18]. The accelerator for channel gating neural networks removes the unnecessary feature map from neural networks [19]. ALAMO generates RTLs for AlexNet or NiN convolutional neural network architectures and allows the exploration of various convolutional neural network structures [20]. VIBNN equips a random number generator for executing a Bayesian neural network in the artificial neural network accelerator [21].

Studies show that lightweight artificial intelligence algorithms and accelerators have a purpose in running artificial intelligence algorithms in an embedded system or mobile device. In actuality, the artificial intelligence accelerator has benefit in power consumption, not computational complexity [22,23]. Therefore, we need an accelerator that supports general artificial intelligence algorithms for benefit of power consumption and computational complexity.

In this paper, we propose the ASimOV framework, which optimizes the artificial intelligence algorithm and generates Verilog HDL code for executing the artificial algorithm in FPGA. The simulator in ASimOV allows us to explore the performance space of artificial intelligence algorithms for problems, and the HDL code generator in ASimOV allows for executing optimized artificial intelligence algorithms at simulation phase in FPGA. To verify ASimOV, we explore the performance space of the k-NN accelerator in the image, speech-recognition dataset, and execute the generated k-NN accelerator optimized in the simulation phase in FPGA.

The rest of this paper is organized as follows. Section 2 describes the AI algorithms. Section 3 describes ASimOV framework that was composed of a simulator and HDL code generator. Section 4 provides the experiment result of the artificial intelligence algorithms’ simulation at various parameter settings. Section 5 summarizes the proposed framework, and presents the future work.

## 2. Background

The parametric method and non-parametric method are methods that estimate probability density function from sampling data. The non-parametric method has two advantages. First, the risk of choosing the wrong parametric assumption is small. Second, it is less sensitive to outliers. The histogram method is one of the representative non-parametric methods. A total number of bins has exponential scaling with D when we divide each variable in a D dimensional space into M bins. Sampled high dimensional data have a property that represents sparse in high dimension space. To estimate the probability density at a particular location, we need data points that lie within some local neighborhood of that point. Therefore, it is hard for that histogram method to apply to high-dimensional data. To overcome this limitation, we can use kernel density estimation using the kernel function. The parameters defining the kernel are the same for all kernels when density estimation is performed through the kernel estimation method. If a large parameter is used, it is excessively smoothed and it is difficult to reveal the original structure of the probability density. If a small parameter is used, the noise is reflected in the probability density for regions with low data density. Therefore, the kernel optimal parameter value of kernel density estimation is bound to be dependent on its location in the data space. This problem can be solved through k-NN, which allows parameters to be adjusted according to the data density. k-NN can be used as a classifier because it can obtain the posterior probability of which class the data belong to by applying the Bayesian Theorem to the density estimation equation of kernel density estimation.

k-NN is a nonparametric method and used for classification or regression problems. During training, k-NN stores all training data. In the process of inference, when new data come in, the distances of the stored data are all compared, and the nearest data point is returned. Therefore, k-NN is a memory-intensive algorithm with fast learning, slow inference, and storing all data. The k-NN classifier selects one category by voting for the selected data candidate group. The k-NN regressor averages and uses the values of the data candidates. Algorithm 1 below shows the pseudocode of the k-NN classifier. The parameters of the k-NN algorithm are divided into two types: the number of neighbors to be searched (k), and the distance measurement method. When the k-value is small, the local characteristics of the data are excessively reflected, and when the k-value is too large, it is over-normalized. For the distance measurement methods such as Euclidean and Manhattan, Mahalanobis correlation distance is used according to the definition of the problem. When trying to classify or regress high-dimensional data, the k-NN algorithm has a limitation in that the average distance between neighboring neighbors and the average distance between all data become similar, resulting in poor prediction performance. To solve this problem, the problem is solved by projecting high-dimensional data in a low-dimensional manner through a dimensional reduction technique such as principal component analysis (PCA) [24,25,26,27].
**Algorithm 1.** Pseudo-code for the k-NN Algorithm.**Input**:
X: training data
Y: class labels of X
x: unknown sample
S: instance set
k: number of nearest neighbor**Output**: predicted class label1://train phase2:**for** i = 1 to length(X) **do**3:
  Store training instance X[i], class label Y[i] into instance set S[i]
4:**end for**5:
6://inference phase7:**for** i = 1 to length(X) **do**8:
  Calculate distance between the unknown sample and the stored training instance d(X[i], x)9:
  Store calculated distance values into D
10:**end for**11:
12:Find k minimum distance values13:Vote which class is the most among k minimum distance values14:
15:Return the class that has been voted by the majority

The k-NN is divided into structureless k-NN, which overcomes memory limitations during learning, and structure-based, which reduces computational complexity during inference. Structure-based k-NN utilizes the tree structure and uses an approach that forms a tree data structure during learning and reduces search time during inference. At this time, the characteristics vary depending on which tree structure is used [28,29,30]. Structureless k-NN is improved from the condensed nearest neighbor, which removes duplicate patterns during training [31,32,33], and the reduced nearest neighbor, which removes even patterns that do not affect the training dataset result [24,34], and WkNN, which adds weight to distance values [35]. Most of the problems that AI algorithms deal with change their approach depending on the dataset. Depending on the dataset, classes can be imbalanced, and there can be a lot of data with similar patterns. Moreover, a certain feature point may contain a lot of meaningful information. When it is solving a specific problem, the AI algorithm is affected by the data characteristics, and this influence also affects the AI accelerator. Therefore, before designing a k-NN accelerator, it is necessary to review and optimize the structure of a k-NN accelerator suitable for a specific problem. It is similar to testing hardware logic in FPGA before application-specific integrated circuit (ASIC) [36,37,38]. Therefore, in order to overcome this problem, this study proposes an ASimOV framework that examines and optimizes various k-NN accelerators to generate HDL code that can be executed directly on the FPGA.

## 3. Methodology

The proposed framework consists of a simulator and generator. As the resource of an embedded system is limited, we analyze how to exploit the resource efficiently by the proposed simulator. Based on the analyzed results, the HDL code generator generates a hardware model with high efficiency of resources, even the limited resource of the embedded system. In this paper, we generate the hardware model by the proposed framework and construct an embedded system by connecting the hardware model to an embedded processor.

### 3.1. ASimOV Framework

The hardware model generated by ASimOV’s HDL generator is designed to support pure k-NN. Therefore, the simulator only supports structureless-based k-NN. This is achieved by injecting custom Python source code into the simulator. When the user navigates through the various parameters related to the design, the user chooses a passive method and automatic method. This changes greatly depending on what type of code the user is using. For the passive method, for example, users can insert custom Python code that analyzes k-NN’s performance after one training and inference and manually changes the parameters. In the case of the automatic method, the user can insert the Python source code which automatically finds the best parameters.

**Figure 1:**
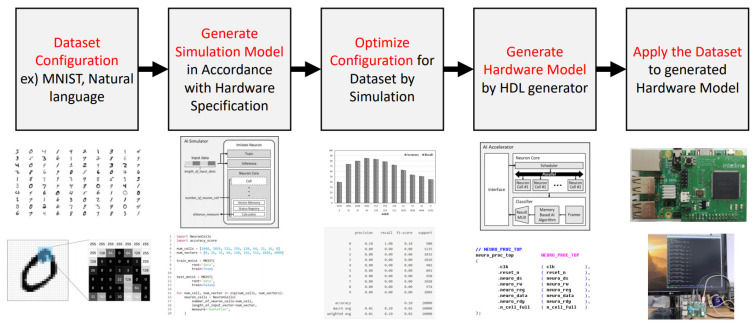
Design sequence of ASimOV framework.

Figure 1 shows the design sequence of the proposed framework. The first line is the explanations of ASimOV framework sequence, the second line is the images for description and the last line is the execution results. Before designing the embedded AI system, the user decides the application and available hardware resources. Testing the embedded AI system by implementing hardware shows high accuracy, but it also requires high testing costs. Moreover, when they change the application of hardware resources, it is inefficient because it needs to be tested again. Therefore, simulating the AI system using the proposed simulator reduces the cost of the system. The user of the ASimOV generates a simulation model that has the same specification as the target embedded system or FPGA. The parameters are determined by the available resources of the target FPGA. In the sequence of generating the simulation model, the user sets the maximum number of neuron cells according to the amount of memory available and the logic size of the FPGA. After the generating simulation model, the user gets an optimized configuration through the repeated simulation. As a result, the ASimOV makes the user get an optimized hardware model for the dataset they use.

For example, we can explore the performance space of artificial intelligence algorithms, given datasets and hardware specs. This exploration has an advantage that reduces exploration cost when we explore different domain problem at the same algorithms. After the simulation, the proposed HDL generator generates a hardware model that changed some components such as memory, given hardware parameters from the simulator. As a result, the proposed framework possibly allows user to use an optimized embedded AI system.

The overall sequence of the framework is as follows. First, the users decide what dataset they use for the embedded system. When the users decided the dataset, users also decide the specification of the embedded system, such as memory for the AI accelerator. The determined size of memory influences the generation of simulation models in the next step. Depending on the size of the memory, the user checks the results of the simulation, depending on the number of possible cells and the amount of memory a cell has. Based on the number of optimized cells and the memory size of the cells from the result of the simulator, users generate a hardware model using the HDL generator.

The last line of Figure 1 is the result of generating an MNIST classification model using the ASimOV framework. First, the user decides the dataset they want to use and the hardware resource they can use. In accordance with the hardware resource, the user makes a simulation model with various configurations of the number of cells and the size of the vector. When the parameters are decided, the simulator finds an optimized configuration of the number of cells and the size of the vector. The simulation result shows all of the configurations of precisions, recalls, and f-1 sources. The user chooses one of the configurations and inputs the configuration data to the HDL generator. The result of the HDL generator generates Intel Quartus Prime 17.1 project, but the user can exploit only the register-transfer level (RTL) codes. If the user uses the Quartus Prime project, the user can exploit the AI accelerator by just compiling and uploading the design on FPGA. The generated AI accelerator is controlled through serial peripheral interface (SPI) communication with the main processor.

### 3.2. AI Simlator

The proposed simulator is designed to emulate the AI algorithms for implementing on hardware. Therefore, the proposed simulator enables the user to construct the optimized embedded AI system for application without implementing the hardware. When the system has already been implemented in the hardware, the performance verification using the simulators has the advantage of reducing the design costs. In this paper, we construct the AI accelerator with a distance-based AI algorithm, k-NN. The simulation consists of Python code and is designed to take advantage of custom Python code. The simulation only provides an interface for training, inference, and HDL generation.

**Figure 2:**
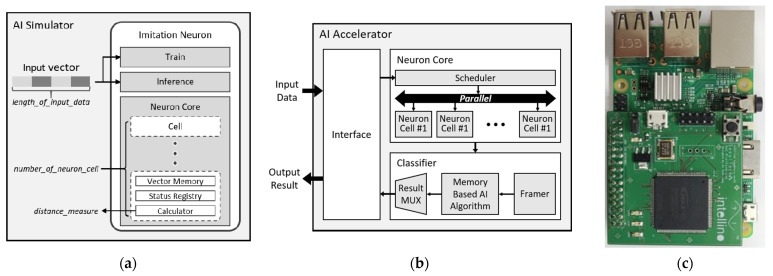
(**a**) Construct of the proposed simulator; (**b**) Architecture of the AI accelerator; (**c**) Prototype board of AI accelerator with embedded processor.

In order to emulate the hardware as much as possible, the proposed simulator is designed to imitate the functionality of modules in the AI accelerator. Figure 2a shows the construct of the proposed simulator and Figure 2b shows the architecture of the AI accelerator. Because optimizing hardware has high cost, the proposed simulator consists of emulating each module of Figure 2b. The proposed simulator generates an imitation neuron, which is responsible for the main function in hardware and conducts a simulation by entering input vectors for learning and inference. The imitate neuron consists of train, inference, and core which is the set of cells. The train and the inference emulate classifier in hardware and the cell emulates the neuron-cell of the neuron core module. The *cell* includes the vector mem. to store training data, a status registry for indicating the status of the neuron, and the calculator for performing distance calculations.

The proposed simulator provides artificial intelligence algorithms based on k-NN. Because the hardware model follows the pure k-NN algorithms, the simulator also follows the pure k-NN algorithms. Therefore, the simulator only applies methods that have not changed operation manner, such as a condensed nearest neighbor, reduced nearest neighbor, and WkNN method. In this paper, we focus on pure k-NN algorithms. When generating an imitate neuron, the AI simulator needs the information about the number of cells to configure neuron core and the size of the input vector to store in the vector mem. The product of the number of cells and the size of the data corresponds to the memory cell of the hardware. Because the number of cells is related with the number of categories and the size of the input vector is related with the detail of data, the neuron core needs to be generated in appropriate configurations in accordance with the application.

At the learning stage of the AI simulator, data are stored in a cell with category information. As the train resizes the data according to the size of vector mem., the proposed simulator enables the user to test the various configuration of memory without additional modification. In the inference stage, the proposed simulator receives the recognition data and calculates the distance with trained data in cells according to information of status registry. The inference returns a category of cells with a minimum distance by comparing all the distance values of the cells.

The learning/inference process of the simulation allows you to find the optimal com-bination of the vector mem. and the number of cells. This optimal combination is passed to the HDL generator to generate the hardware model.

### 3.3. HDL Code Generator

The HDL code generator generates a hardware model based on the optimized configuration obtained from the AI simulator. The architecture of the generated hardware model is shown in Figure 2b. The proposed AI accelerator consists of the interface module, neuron core module, which is set of neuron cells, and the classifier module. The interface module communicates with embedded processor to get the learning data and to send the recognition result by the serial communication. The classifier module determines the result category in accordance with the selected AI algorithm.

During the training, the entered data are stored in each cell by the scheduler with the category data. Each cell has memory for storing input data. The number of cells and the size of memory are generated in an optimized number and size obtained from the simulator, and data are stored directly in memory through the scheduler. When performing the inference, the recognition data are sent to all activated cells simultaneously and distance computation results are sent to the classifier. The classifier transmits the results category according to the algorithm used. The framer assists the AI algorithm by organizing the distance result and the category of the cells. In this paper, we use the k-NN algorithm that determines the result category with the shortest distance of neuron cell. When the inference is over, the result MUX transmits the result data to the interface module to send to the embedded processor.

In order to verify the proposed framework, we exploited an FPGA of the Intel MAX10 series and designed prototype board shown in Figure 2c. The proposed HDL code generator generates not only the HDL codes, but also the project of Intel Quartus Prime 17.1, including the FPGA information. The memory model of the HDL generator is based on 1-port RAM that is provided by the Quartus Prime IP. The RTL except memory is not tied to such vendors, therefore users can implement the HDL codes by changing the memory model they want to use. For the experiments of proposed HDL codes, we adopt ARM Cortex-A series as the main processor and control the processor using C programing. The main processor is not included.

## 4. Experiments

In order to validate the proposed framework, we used image datasets and speech datasets. The image model is tested with MNIST, Fashion MNIST, CIFAR-10, and STL-10 [39,40,41,42]. The speech model is tested with the google speech command dataset and VCTK, with three different pre-processing methods [43,44]. We simulated 16 KB and 64 KB of memory for the proposed framework. In this paper, 3-fold cross-validation was used to evaluate the algorithm performance in each dataset. Since the simulation of the proposed framework performs the same functions as the implemented hardware, we did not add the hardware result separately. The results in Table 1, Table 2, Table 3 and Table 4 are the same for both software and hardware.

### 4.1. Image Dataset

For the test of the proposed framework, we used MNIST, Fashion MNIST, CIFAR-10, and STL-10. Table 1 and Table 2 show the accuracy of the simulation and generated hardware by framework with 16 KB memory and 64 KB of memory, respectively. The MNIST, Fashion MNIST, CIFAR-10, and STL-10 show the best result at 64 bytes with 256 cells, 128 bytes with 128 cells, 16 bytes with 1024 cells, and 64 bytes with 256 cells respectively. The vector length of the best result in MNIST, CIFAR-10 and STL-10 is same regardless of the total memory size. However, the best configuration of Fashion MNIST is changed from 128 bytes with 128 cells to 64 bytes with 1024 cells when the total system memory was 64 KB. The simulation results indicate that when the total memory size is changed, the performance of the distance-based algorithms changes according to not only the vector size but also the number of cells. Therefore, the proposed framework optimizes and improves the performance of the AI accelerator.

### 4.2. Speech Dataset

Table 3 and Table 4 show the accuracy of the simulation and generated hardware by the framework using speech models with 16 KB memory and 64 KB memory, respectively. For better performance on speech recognition, we exploited short-time Fourier transform (STFT), Mel spectrum, and Mel-Frequency Cepstral Coefficient (MFCC). The google speech command result using STFT of 16 KB memory model shows the highest performance at 87.4% with 16 bytes vector length and 1024 number of neuron cells. However, the 64 KB memory model has the best performance at 90.7% on 32 bytes vector length and 2048 neuron cells. The increase in the number of neuron cells increases 2.8%p. The VCTK also shows the highest performance at different configuration as memory increases. Therefore, the ASimOV framework has more chances to increase performance with various memory configurations.

### 4.3. Other Method

There are two kinds of k-NN. One is called structure method, which reduces computational complexity during inference using tree data structure. The other is a structureless method that overcomes memory limitations during training by removing similar data. Hardware that supports the structure approach must be suitable for tree-like data structures. However, as there is no structured way to overcome the memory limit by removing similar data, this does not require any hardware changes.

Hardware has a limitation in which it is difficult to change the internal structure dynamically as needed. Therefore, ASimOV that has limitations equally with hardware supports the structureless method, which does not change only the internal structure. Table 5 shows the accuracy of the simulation by structure les method with 16 KB memory in the MNIST dataset.

The weighted, calculated distance of weighted k-NN achieves 81% at vector length 32, 64, with the number of cells at 512, 256. Condensed k-NN, which removes similar data, achieves 78% accuracy at vector length 128, with the number of cells at 128. Pure k-NN achieves 82% at vector length 64, with the number of cells at 256.

The weighted k-NN method showed 81% accuracy with a vector length of 32 and 512 cells, which means that the weighted k-NN operates at a lower resolution than pure k-NN. Condensed k-NN had a 4% performance drop compared to pure k-NN, but it is not clear what this means.

**Figure 3:**
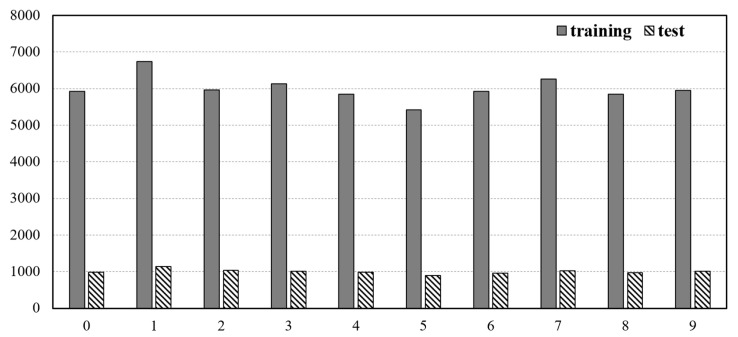
MNIST dataset distribution.

Condensed k-NN is a down sampling technique that removes unnecessary data or balances data in unbalanced data. Condensed k-NN is difficult to obtain meaningful results when there is minimal data for classification. To confirm this, the distribution of the MNIST dataset was checked, and Condensed k-NN was applied to all MNIST datasets without considering hardware specifications. Figure 3 shows the distribution of the MNIST dataset, and Table 6 shows the accuracy of Condensed k-NN and pure k-NN for the entire MNIST dataset.

Figure 3 describes the MNIST data as uniform data. The number of cells of the condensed k-NN in Table 6 is the same as the number of remaining MNIST datasets after unnecessary data has been removed with the condensed k-NN. As a result of comparing the performance of condensed k-NN and k-NN, it can be seen that condensed k-NN learns with about eight times less data, and that the accuracy is 10% lower when compared with k-NN that has learned the entire data. Through this, it can be confirmed that condensed k-NN is a technique that can be used when a trade-off between the number of datasets and performance is required.

### 4.4. Power Consumtion

AI algorithms utilize external computing devices such as GPUs. A GPU consumes more than 100 W of power. The power consumption of external computing devices such as GPUs makes it difficult to apply AI algorithms to embedded systems or mobile devices.

In order to confirm that the ASimOV proposed in this paper is more efficient in power consumption compared to other AI algorithms, the power consumption of the ASimOV simulator, hardware, and AI model was measured and compared. Table 7 shows the power consumption of the simulator, hardware and AI model of ASimOV.

The experimental environment for measuring the power consumption of ASimOV is divided into a software measurement environment and a hardware measurement environment. Since the simulator and AI model are software, power consumption was measured in a desktop environment. At this time, the desktop configuration used is Intel i9-11900 K, 94.2 GB Memory, Nvidia RTX-3090 (Nvidia Corporation, Santa Clara, CA, USA). In the desktop environment, the power usage was estimated using the powertop application of Linux. The power consumption of hardware was measured by measuring the current entering the accelerator and multiplying the operating voltage. To measure and compare the power consumption of ASimOV, the simulator and hardware used the k-NN algorithm in the MNIST dataset, and the AI model used LeNet, a representative convolutional neural network.

As a result, it was confirmed that Simulator 1.54 W, Hardware 0.77 W, AI Model 8.03 W was used. Through this, it was confirmed that the hardware generated by ASimOV uses less power compared to the AI model.

## 5. Conclusions

In this paper, we propose ASimOV, an end-to-end framework that allows optimization of the artificial intelligence accelerator in the specific dataset. The ASimOV framework consists of two parts: an AI simulator and an HDL code generator. The AI Simulator in ASimOV finds the optimal parameter for aiming maximum performance such as accuracy in the parameter search space of the artificial intelligence accelerator and algorithms. The HDL code generator in ASimOV generates a hardware model using optimal parameters of the artificial intelligence accelerator and algorithm. The hardware model implements functional testing purposes in FPGA. The ASimOV reduces the cost of design of the artificial intelligence accelerator in the specific dataset, as a provided semi-automatic procedure with a simulator and HDL code generator. In Section 4, we perform functional verification of ASimOV in small artificial intelligence algorithms, such as character recognition, clothing recognition, speech recognition, etc. For the AI accelerators using k-NN, ASimOV shows up to 90.7% performance in total with 64 KB memory. In future work, we will add various artificial intelligence algorithms such as a support-vector machine, decision tree, and analysis or preprocessing tools such as principal component analysis for ASimOV. From the viewpoint of accelerator architecture, we will research multi-core accelerator architecture. This architecture needs to be managed, and that management to be scheduled to single-core due to task priority. We expect that ASimOV facilitates a fast fail in simulation environments, and an easy-to-apply artificial intelligence accelerator in various domains.

## Figures and Tables

**Table 1:** Simulation result of image datasets with 16 KB of memory.

Vector Length	Number of Cell	MNIST	Fashion MNIST	CIFAR-10	STL-10
8	2048	46.3%	47.6%	16.5%	23.6%
16	1024	79.0%	72.9%	**22.1%**	22.3%
32	512	78.9%	75.8%	21.7%	23.0%
64	256	**83.9%**	76.6%	20.3%	**23.8%**
128	128	82.3%	**77.4%**	20.9%	22.8%
256	64	77.9%	72.1%	18.9%	19.8%
512	32	69.0%	68.9%	15.9%	17.8%
1024	16	56.0%	58.8%	16.5%	17.2%
2048	8	53.3%	48.9%	13.2%	13.4%

**Table 2:** Simulation result of image datasets with 64 KB of memory.

Vector Length	Number of Cell	MNIST	Fashion MNIST	CIFAR-10	STL-10
8	8192	47.7%	48.3%	16.2%	17.1%
16	4096	81.1%	75.4%	**24.1%**	24.8%
32	2048	83.6%	78.7%	23.1%	25.2%
64	1024	**90.3%**	**80.0%**	23.2%	**25.5%**
128	512	89.6%	79.2%	22.0%	25.0%
256	256	85.5%	77.2%	20.5%	24.8%
512	128	81.5%	76.4%	20.7%	22.7%
1024	64	77.3%	73.6%	19.0%	19.7%
2048	32	68.3%	69.8%	15.8%	17.9%

**Table 3:** Simulation result of speech datasets with 16 KB of memory.

Vector Length	Number of Cell	Google Speech Command			VCTK		
		STFT	MEL	MFCC	STFT	MEL	MFCC
8	2048	80.5%	59.1%	51.8%	57.3%	40.0%	48.7%
16	1024	**87.4%**	**86.7%**	62.8%	82.7%	76.7%	66.7%
32	512	87.2%	86.6%	63.6%	82.7%	**82.7%**	**74.0%**
64	256	84.1%	83.6%	65.6%	**86.7%**	80.0%	61.3%
128	128	84.4%	82.1%	**66.0%**	83.3%	77.3%	62.7%
256	64	81.9%	78.9%	63.5%	79.3%	76.0%	61.3%
512	32	79.5%	74.2%	61.8%	70.0%	62.7%	53.3%
1024	16	74.4%	69.7%	65.1%	76.7%	56.7%	54.7%
2048	8	69.6%	66.1%	59.1%	53.3%	52.7%	49.3%
4096	4	66.7%	59.5%	51.0%	42.0%	43.3%	39.3%

**Table 4:** Simulation result of speech datasets with 64 KB of memory.

Vector Length	Number of Cell	Google Speech Command			VCTK		
		STFT	MEL	MFCC	STFT	MEL	MFCC
8	8192	80.9%	56.7%	51.2%	57.3%	40.0%	48.7%
16	4096	90.2%	**90.5%**	65.4%	82.7%	76.7%	66.7%
32	2048	**90.7%**	89.7%	67.0%	79.3%	86.0%	68.7%
64	1024	89.5%	89.3%	68.0%	84.0%	**77.3%**	68.0%
128	512	88.3%	88.4%	**74.6%**	**84.0%**	76.7%	66.7%
256	256	85.4%	85.3%	73.8%	83.3%	80.7%	**71.3%**
512	128	84.8%	84.4%	69.2%	82.0%	79.3%	67.3%
1024	64	82.1%	78.0%	74.0%	76.7%	70.7%	67.3%
2048	32	79.5%	73.3%	72.6%	68.0%	57.3%	60.0%
4096	16	74.0%	69.6%	65.3%	62.7%	56.0%	54.0%

**Table 5:** Simulation result of structureless method with 16 KB of memory in MNIST datasets.

Vector Length	Number of Cell	Condensed k-NN	Weighted k-NN	k-NN
8	2048	24%	27%	28%
16	1024	62%	74%	73%
32	512	66%	**81%**	80%
64	256	68%	**81%**	**82%**
128	128	**78%**	73%	76%
256	64	48%	63%	69%
512	32	36%	53%	60%
1024	16	29%	44%	49%
2048	8	13%	31%	19%

**Table 6:** Simulation result of Condensed k-NN, k-NN in MNIST datasets.

Vector Length	Number of Cell	Condensed k-NN	k-NN
128	7693	86%	-
128	60,000	-	96%

**Table 7:** Power consumption of Asimov simulator, hardware and AI model.

	Simulator	Hardware	AI Model (LeNet)
power consumption	1.54 W	0.77 W	8.03 W
